# Supplementary material for: Paternal hypoxia exposure primes offspring for increased hypoxia resistance
Source: BMC Biol. 2022 Aug 30;20:185. doi: 10.1186/s12915-022-01389-x (PMC9426223; doi:10.1186/s12915-022-01389-x)
Supplement: Supplementary file 2 — Additional file 2: Table S2. Read counts mapping to four hemoglobin genes. [file 12915_2022_1389_MOESM2_ESM.docx]

**Table S2**: **Reads** (**counts per million) of four haemoglobin genes located on a small region of chromosome 3**. * genes are not significantly differentially expressed but show strong expression in samples H1 and H2 who were the most resilient to loss of equilibrium in hypoxic environment. Note that there are two versions of hbba1 due to genome duplication, and they are physically separated on chromosome 3.

| **Ensembl ID** | **Gene name** | **C1** | **C2** | **C3** | **H1** | **H2** | **H3** |
| --- | --- | --- | --- | --- | --- | --- | --- |
| **ENSDARG00000079078** | *hbz* | 0.06 | 0.21 | 0.85 | 15.15 | 85.63 | 1.43 |
| **ENSDARG00000097011** | *hbaa1* | 0.12 | 0.07 | 1.11 | 44.79 | 104.53 | 0.95 |
| **ENSDARG00000097238** | *hbba1** | 0.00 | 0.00 | 0.13 | 0.60 | 22.19 | 0.07 |
| **ENSDARG00000089087** | *hbba1** | 0.00 | 0.00 | 0.52 | 22.76 | 71.35 | 0.54 |
